# Structural Features of *Clostridium botulinum* Neurotoxin Subtype A2 Cell Binding Domain

**DOI:** 10.3390/toxins14050356

**Published:** 2022-05-19

**Authors:** Kyle S. Gregory, Tejaswini B. Mahadeva, Sai Man Liu, K. Ravi Acharya

**Affiliations:** 1 Department of Biology and Biochemistry, University of Bath, Claverton Down, Bath BA2 7AY, UK; kg540@bath.ac.uk (K.S.G.); tejaswini.brahmadevarahalli.mahadeva@bath.edu (T.B.M.); 2 Protein Sciences Department, Ipsen Bioinnovation Limited, 102 Park Drive, Milton Park, Abingdon OX14 4RY, UK; sai.man.liu@ipsen.com

**Keywords:** botulinum neurotoxin, cell-binding domain, subtype A2, crystal structure, ganglioside binding, redox switch

## Abstract

Botulinum neurotoxins (BoNT) are a group of clostridial toxins that cause the potentially fatal neuroparalytic disease botulism. Although highly toxic, BoNTs are utilized as therapeutics to treat a range of neuromuscular conditions. Several serotypes (BoNT/A-/G, /X) have been identified with vastly differing toxicological profiles. Each serotype can be further sub-categorised into subtypes due to subtle variations in their protein sequence. These minor changes have been attributed to differences in both the duration of action and potency for BoNT/A subtypes. BoNTs are composed of three domains—a cell-binding domain, a translocation domain, and a catalytic domain. In this paper, we present the crystal structures of the botulinum neurotoxin A2 cell binding domain, both alone and in complex with its receptor ganglioside GD1a at 1.63 and 2.10 Å, respectively. The analysis of these structures reveals a potential redox-dependent Lys-O-Cys bridge close to the ganglioside binding site and a hinge motion between the H_CN_ and H_CC_ subdomains. Furthermore, we make a detailed comparison with the previously reported H_C_/A2:SV2C structure for a comprehensive structural analysis of H_C_/A2 receptor binding.

## 1. Introduction

Botulinum neurotoxins (BoNTs), although highly toxic, are now routinely used as therapeutics with over 100 medicinal applications [1]. They function by cleaving soluble *N*-ethylmaleimide-sensitive factor attachment protein receptor (SNARE) proteins, halting the release of acetylcholine at the neuromuscular junction (NMJ) resulting in flaccid paralysis [2]. This makes them exceptional candidates for the treatment of a range of neuromuscular disorders, and advancements in biotechnology have resulted in the further expansion of their therapeutic potential [3,4]. Several immunologically distinct BoNT serotypes produced by *Clostridium botulinum* (BoNT/A-/G, /X) have been identified [5], with a growing number of subtypes (e.g., BoNT/A1, /A2, and /A3) that arise due to subtle variations in amino acid sequences [6]. Furthermore, BoNT-like molecules have also been identified in non-clostridial species, such as *Weissella oryzae* [7], *Enterococcus faecium* [8,9], and *Paraclostridium bifermentans* [10]. The substantial number and varying toxicological profiles of BoNT serotypes and subtypes provide opportunities of developing fine-tuned medicines for specific applications.

BoNTs are expressed as a single polypeptide chain that must be cleaved post-translationally into the active di-chain, consisting of an N-terminal Light Chain (LC) and a C-terminal Heavy Chain (HC) held together by a single disulphide bond. The LC is a Zn^2+^-endopeptidase domain, whereas the HC comprises two domains—an N-terminal translocation domain (H_N_) and a C-terminal cell binding domain (H_C_) [11]. Understanding how each domain contributes to toxicity will aid in the development of BoNT molecules for medicinal applications. For BoNT/A, the H_C_/A binds to both a ganglioside (e.g., GD1a) and synaptic vesicle protein 2 (SV2C) [12] at the nerve terminals of the neuromuscular junction. Upon binding, the molecule is internalized by endocytosis. Subsequently, in response to the acidic environment of the endosome, the H_N_/A undergoes conformational change that results in the translocation of the LC/A across the endosomal membrane [13,14,15,16]. Once in the cytosol, LC/A is released by reduction in the disulphide bond [17] so that it may cleave its target SNARE protein, a 25 kDa Synaptosomal-Associated Protein (SNAP-25) [18].

Considering that the H_C_ domain has been shown to contribute to BoNT potency [19], the determination of the precise molecular interactions across the BoNT-ganglioside interface, as well as the associated conformational changes that occur upon binding, may reveal features that contribute to variation in toxicity across BoNT serotypes and subtypes [20,21,22]. Previous structures (PDB: 5MOYand 6ES1) of H_C_/A2 in complex with its protein receptor, SV2C (H_C_/A2:SV2C), revealed conformational changes mostly to SV2C itself [23,24]. In this paper, we present the crystal structures of H_C_/A2 alone and also in complex with GD1a receptor oligosaccharide (H_C_/A2:GD1a) and describe the key residues involved with ganglioside binding, and the conformational changes that occur. Furthermore, we observed a ‘significant’ hinge motion between the H_CC_ and H_CN_ subdomains when compared to reported structures H_C_/A2:SV2C, and note a potential redox-dependent Lys-O-Cys bridge close to the ganglioside binding site (GBS).

## 2. Results and Discussion

### 2.1. Structure of H_C_/A2

**Figure 1:**
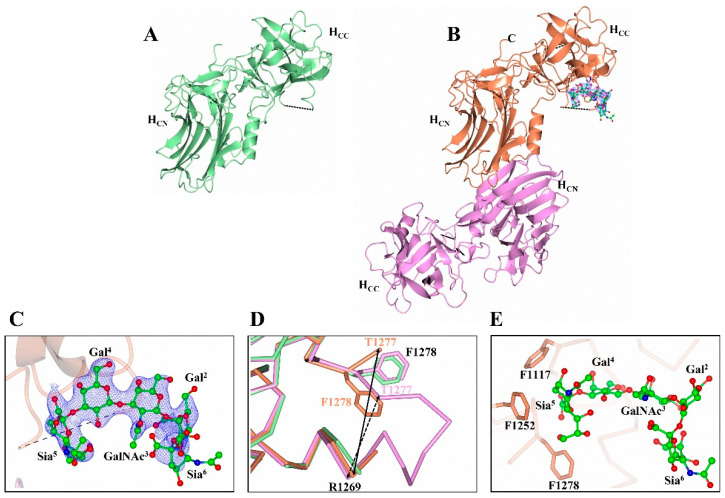
Structure of botulinum neurotoxin A2 cell-binding domain. (**A**) Crystal structure of H_C_/A2 (pale green). (**B**) Crystal structure of H_C_/A2 in complex with GD1a (H_C_/A2:GD1a). (Molecule B is displayed in orange, and molecule A in magenta.) (**C**) Electron density map (2Fo-Fc) of GD1a contoured to 1 σ level. (**D**) Opening of the R1269-T1277 loop upon binding, and associated flip of F1278. The difference in Cα distance between R1269 and T1277 of molecule A and B is highlighted by a solid and dotted arrow, respectively. (**E**) Hydrophobic pocket formed by F1117, F1252, and F1278 that binds GD1a (ball and stick). Dashed lines indicate unmodelled regions.

The structure of H_C_/A2 was determined to a resolution of 1.63 Å (Table 1) by molecular replacement. It has an N-terminal β-jelly roll fold and a C-terminal β-trefoil (Figure 1A) that is consistent with other H_C_/A subtypes [25,26,27]. The quality of the electron density map is good throughout except for a small loop region (Arg 1269-Phe 1277). This loop (which is conserved across all BoNT/A subtypes) precedes the ganglioside binding site (GBS) and appears to be disordered for other H_C_/A subtypes [28,29]. This is likely due to the inherent flexibility of this loop so that it can accommodate ganglioside binding.

### 2.2. Structure of H_C_/A2 in Complex with GD1a Receptor Ganglioside

**Figure 2:**
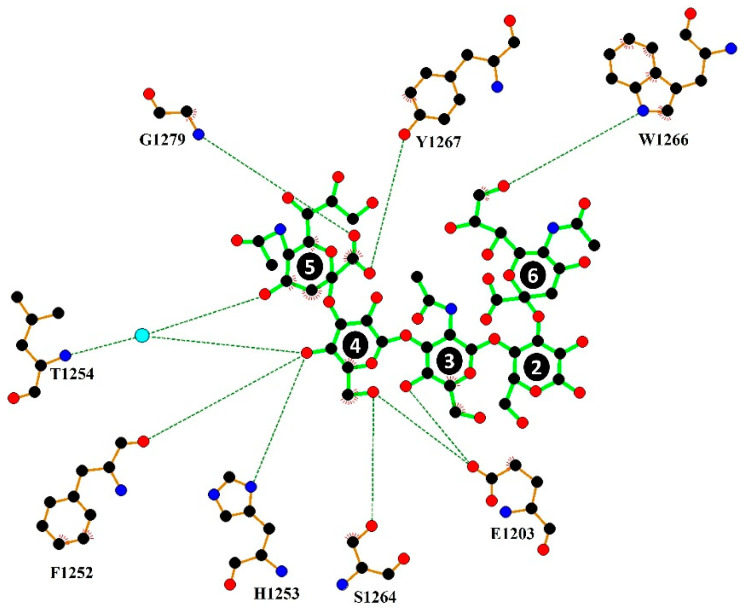
Ligplot+ representation of H_C_/A2:GD1a interactions. Hydrogen bonding interactions are represented by a dotted line. The cyan sphere indicates a water-molecule-mediated interaction. The first glucose was not modelled due to weak electron density, 2 & 4 = galactose, 3 = *N*-acetylgalactosamine 5 & 6 = sialic acid.

The structure of H_C_/A2:GD1a complex was solved by molecular replacement to 2.1 Å (Table 1) with two molecules (A and B) in the asymmetric unit (Figure 1B). Clear positive difference map electron density was observed at the GBS of molecule B that could be readily modelled as GD1a, whereas for molecule A, the GBS is inaccessible to GD1a due to crystal packing. Therefore, the H_C_/A2:GD1a asymmetric unit contains both the GD1a-bound and unbound states of H_C_/A2. A total of 5/6 monosaccharides are clearly defined by the electron density (Figure 1C). GD1a forms a total of 10 hydrogen bonding interactions to H_C_/A2, two of which are water-mediated (Figure 2). This binding mode is identical to what was observed in H_C_/A3, with the exception of Trp 1266, which binds Sia^6^ in H_C_/A2:GD1a, but is unmodelled in H_C_/A3:GD1a [28].

The superimposition of molecule ‘A’ and ‘B’ (for C_α_ atoms) yields an RMSD of 0.47 Å, indicating that the overall structure of the molecule does not change upon binding GD1a. However, the loop spanning residues 1269–1277 appears to widen upon GD1a binding—the distance between the C_α_ atoms of residues 1269 and 1277 is ~4.5 Å greater in molecule B than that in molecule A (Figure 1D). This change in loop positioning is accompanied by the rotation of Phe 1278 towards the GBS, a feature that has been observed previously in the H_C_/A3:GD1a, H_C_/A4:GD1a, and H_C_/A5:GM1b structures [26,28]. This residue, along with Phe 1117 and Phe 1252, forms a hydrophobic pocket occupied by Sia^5^ (Figure 1E).

### 2.3. H_C_/A2 Is Primed for Receptor Binding

**Figure 3:**
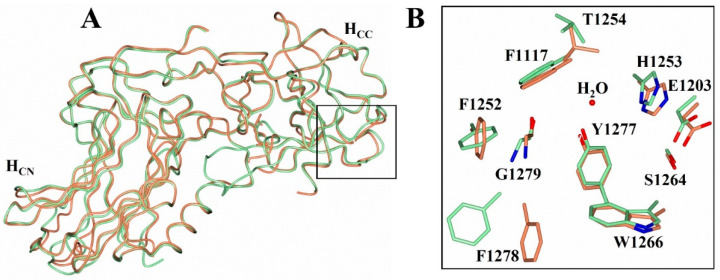
Structural comparison of H_C_/A2 and H_C_/A2:GD1a. (**A**) Superimposition of C_α_ atoms of H_C_/A2 (pale green) and H_C_/A2:GD1a (orange). The ganglioside binding site (GBS) is highlighted by the box. (**B**) Residues comprising the GBS before (pale green) and after binding to GD1a (orange).

**Figure 4:**
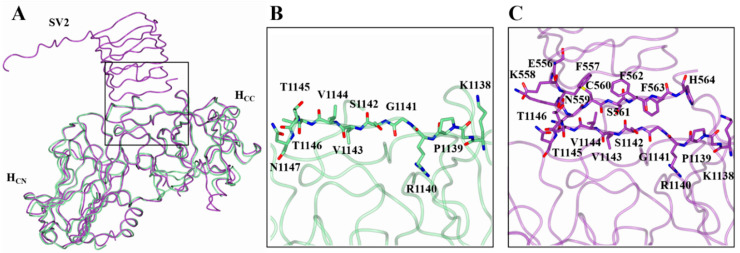
Structural comparison of H_C_/A2 and H_C_/A2:SV2C. (**A**) Global superimposition of C_α_ atoms of H_C_/A2 (pale green) and H_C_/A2:SV2C (purple; PDB: 5MOY). (**B**) Residues of the H_C_/A2 SV2C binding site, indicating the position of key residues (sticks) prior to binding. (**C**) Residues of the H_C_/A2 SV2C binding interface, indicating the position of key residues (sticks) after binding.

The conformational changes that occur with H_C_/A2 upon the binding of GD1a are highlighted in Figure 3. The residues Phe 1252 and Phe 1272 moved towards the GBS and contribute to a hydrophobic patch, while the movement of His 1253 can be attributed to the formation of a hydrogen bond with Gal^4^ (Figure 3B). Compared to a previous structure of H_C_/A2 in complex with SV2C (PDB: 5MOY [23]), the superimposition of C_α_ atoms gave an RMSD of 0.96 Å, indicating that the structure does not drastically alter conformation upon SV2C binding (Figure 4A). There are few changes that occur at the residue level across the H_C_/A2:SV2C interface upon binding (Figure 4B,C); however, there are noticeable differences in the conformation of two loop regions at residues 1164–1172 and 1225–1236. For the former loop, the differences are due to crystal packing, whereas for the latter loop, the variation is likely due to the inherent flexibility as residues 1228 and 1229 could not be modelled. Similar findings were also observed with another reported structure of H_C_/A2:SV2C (PDB: 6ES1 [24]) (not shown). Taken together, the analysis of the ganglioside and SV2C binding sites suggest H_C_/A2 is primed to bind its receptors, requiring minimal conformational change.

### 2.4. Hinge Motion between the H_CN_ and H_CC_ Subdomain

**Figure 5:**
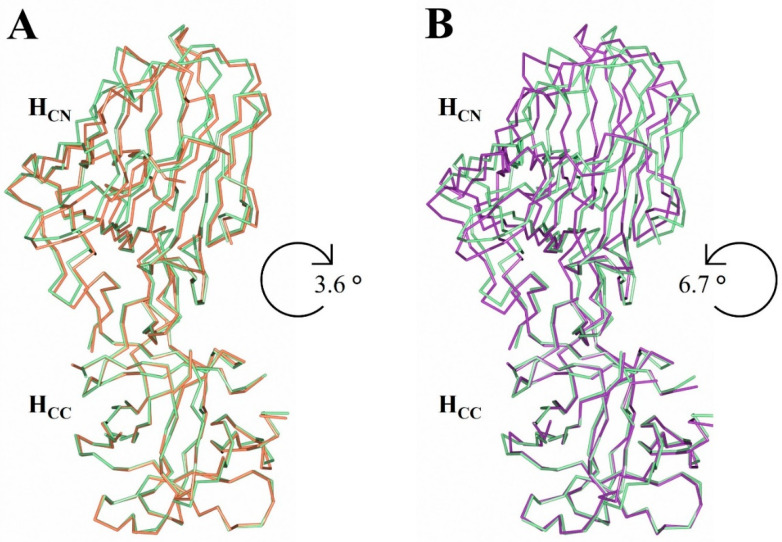
Hinge motion between the H_CN_ and H_CC_ subdomain. Superimposition of the H_CC_ subdomain of H_C_/A2 alone (pale green) with H_C_/A2 in complex with GD1a (orange) (**A**) or SV2C (purple; PDB: 5MOY) (**B**) indicates the presence of a hinge motion between the H_CC_ and H_CN_ subdomains.

The superimposition of H_C_/A2:GD1a and H_C_/A2:SV2C structures with the H_C_/A2 structure revealed a slight misalignment across the entirety of the C_α_ trace (RMSD values of 0.79 Å and 0.99 Å, respectively). The superimposition of just the C-terminal subdomains (H_CC_) showed a nearly identical alignment, with the N-terminal subdomain (H_CN_) rotated out of position relative to each other (Figure 5). Using DynDom (which estimates domain motions in proteins) [30], the H_CN_/A2 subdomain appears to rotate 3.6° in one direction when bound to GD1a (Figure 5A), but 6.7° in the other direction when bound to SV2C (Figure 5B). This hinge-like motion is not believed to facilitate SV2C receptor binding because it does not alter the position of the key binding residues. However, considering that the H_CC_ subdomain is responsible for anchoring BoNT to the NMJ cell membrane, it is possible that the hinge between H_CC_ and H_CN_ may aid in the orientation of the H_N_ and LC towards the membrane in preparation for translocation, which is consistent with what has been proposed previously [31].

### 2.5. Lys 1236-X-Cys 1280 Bridge near the GBS

**Figure 6:**
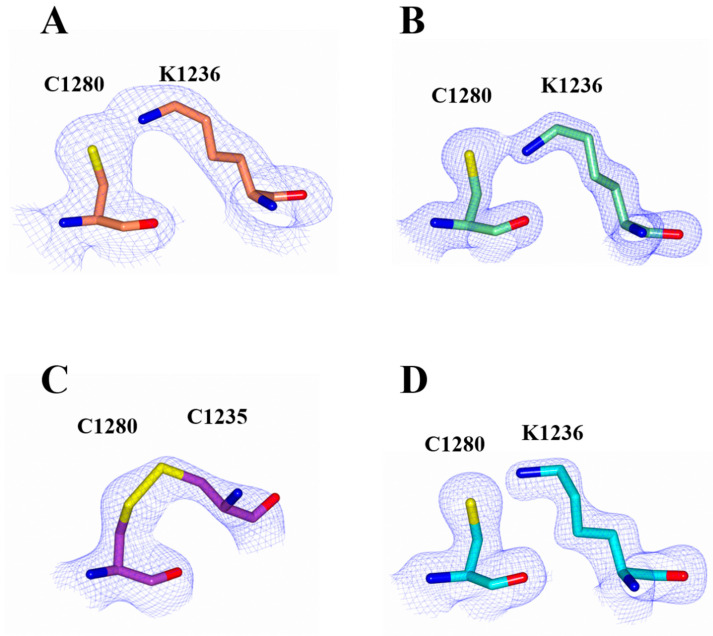
Lys 1236-X-Cys 1280 Bridge. (**A**) 2Fo-Fc electron density (contoured to 1 σ) was observed between C1280 and K1236 of H_C_/A2 when bound to GD1a (H_C_/A2:GD1a) (**A**) and alone (H_C_/A2) (**B**), which indicated the presence of a Lys-X-Cys bridge. When bound to SV2C, however, one structure (PDB: 6ES1) showed the formation of a disulphide bond between C1280 and C1235 (**C**) and another (PDB: 5MOY) showed no electron density between these two residues (**D**).

In both the H_C_/A2 and H_C_/A2:GD1a structures, clear electron density was observed between Lys 1236 and Cys 1280 (Figure 6A,B) indicating the presence of an unusual covalent bridge between the two residues. A similar observation was made in the structure of H_C_/A5 involving equivalent residues [27]. The Lys-X-Cys bridge is a recently reported interaction that may be a widespread phenomenon in many protein structures [32,33]. There are two possible bridging atoms where X is either an O or a C (as a methylene group, CH_2_). The formation of an -O- bridge occurs via the spontaneous oxidation of the cystine sulfhydryl group in the absence of reducing agents, whereas the formation of a -CH_2_- bridge has been suggested to occur by the reaction of a Lys with CO_2_ or CH_2_O [32,34].

**Figure 7:**
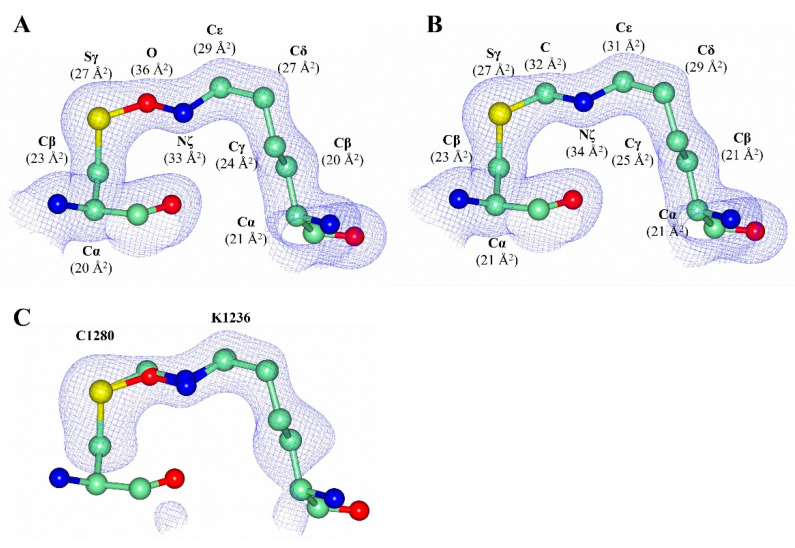
Analysis of the Lys 1236-X-Cys 1280 bridge in H_C_/A2 (present structure). (**A**) The 2Fo-Fc map (contoured at 1 σ level) of Lys-X-Cys modelled as Lys-O-Cys; the B-factors for each atom across the bridge are displayed. (**B**) The 2Fo-Fc map (contoured at 1 σ level) of Lys-X-Cys modelled as Lys-CH_2_-Cys; the B-factors for each across the bridge are displayed. (**C**) Omit map for Lys 1236 and Cys 1280 side chains, contoured at 3 σ level.

Both possibilities were modelled into the electron density of the H_C_/A2 structure and refined (Figure 7A,B). However, it was not possible to determine the identity of the bridging atom; crystallographically, both were equally possible (Figure 7A–C). This illustrates the difficulty in determining the precise nature of the bridging atom, and there appears to be a divided debate on this topic [35].

On the other hand, the previously reported crystal structures of H_C_/A2 bound to SV2C showed either the formation of a disulphide bond between Cys 1280 and Cys 1235 instead (Figure 6C), or no interaction between the two residues (Figure 6D). Interestingly, both of these structures (PDB: 6ES1 and 5MOY, respectively) were determined from crystals grown under reducing conditions, in contrast to the present structure of H_C_/A2 where crystals were grown under non-reducing conditions. This indicates that the formation of the Lys-X-Cys bridge may be dependent on the redox environment. This type of redox-dependency has been observed previously in the transaldolase enzyme from the *Neisseria gonorrhoeae* bacterium, where a Lys-O-Cys bridge serves as an allosteric redox switch [36]. 

Further, a recent report based on a systematic study on the presence of Lys-Cys bridges in protein structures revealed that oxygen is the most likely bridging atom [33]; therefore, we modelled this atom in the final deposited coordinates.

**Figure 8:**
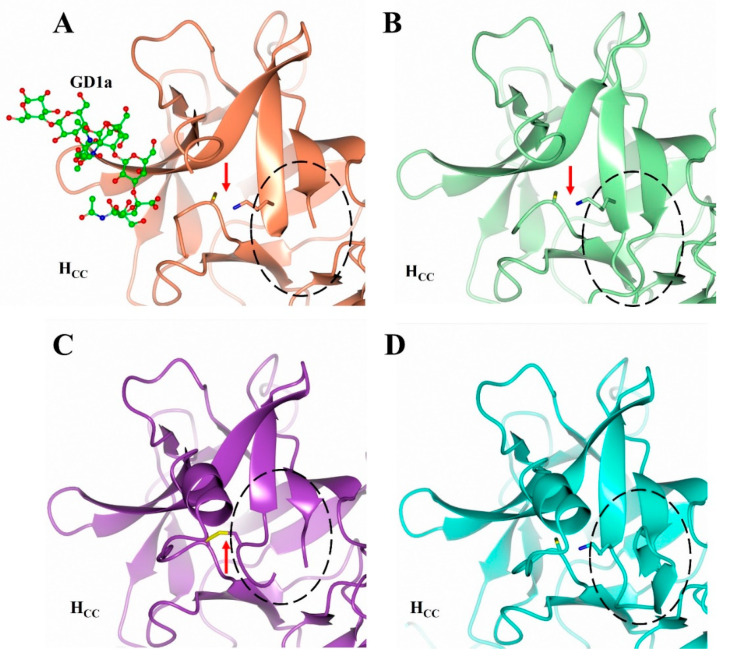
Conformational changes associated with the Lys-X-Cys bridge. Comparison of the structure of the 1220–1240 β-hairpin of H_C_/A2 alone (**B**), in complex with GD1a (**A**), and bound to SV2C ((**C**), PDB: 5MOY; and (**D**), PDB: 6ES1). The 1224–1236 loop is highlighted by the dotted circle, and the red arrow indicates the location of the bridging interaction (either Lys-X-Cys or Cys-Cys).

Although the biological relevance of this Lys 1236-O-Cys 1280 bridge in the present H_C_/A2 structure is currently unknown, it is interesting to note that both these equivalent residues are conserved in all subtypes of BoNT/A, and that they are situated in a dynamic region of the protein close to the GBS. Cys 1280 is located close to Phe 1277, whose sidechain is known to flip orientation upon ganglioside binding, and Lys 1236 is positioned within a β-hairpin (residues 1220–1240) that appears to possess a flexible loop based on the lack of electron density for residues 1224–1236 (Figure 8). Further investigation will be required to confirm the identity and biological function (if any) of this Lys-O-Cys bridge.

## 3. Conclusions

The crystal structures of H_C_/A2 alone and in complex with GD1a reveal a total of 9 residues that form 10 hydrogen bonding interactions with the sugar moiety, accompanied by a conformational change of a loop (residues 1269–1277) located near the GBS. Furthermore, structural comparison with H_C_/A2 bound to its protein receptor, SV2C, revealed features not previously reported in the literature. The H_CN_ and H_CC_ subdomains appear to rotate about a common hinge position depending on which receptor molecule H_C_/A2 binds—ganglioside or SV2C. This motion may be involved in orienting the translocation domain towards the cell surface following dual-receptor-initiated endocytosis. We also note the presence of a Lys 1236-O-Cys 1280 bridge in the two crystal structures of H_C_/A2 presented here that is located on a loop near the GBS. The biological significance of both the hinge and Lys-O-Cys bridge is unknown and requires further investigation. This information might be valuable in the bioengineering and manufacture of BoNT/A subtypes for enhanced therapeutic applications.

## 4. Materials and Methods

### 4.1. Expression and Purification of H_C_/A2

The pJ401 vector containing H_C_/A2 (BoNT/A2 residues 871–1296) was transformed into One Shot BL21 (DE3) Star competent cells (Thermo Fisher Scientific, Loughborough, UK), as previously described [37]. Cultures were grown in TB at 37 °C until an OD_600_ of 0.6, and protein expression was induced with 1 mM IPTG at 16 °C for at least 16 h. Cells were lysed in 50 mM Tris pH 7.4, 20 mM imidazole, and 0.5 M NaCl. H_C_/A2 was captured by Ni^2+^ affinity chromatography and eluted with 0.5 M imidazole in 50 mM Tris pH 7.4, 0.5 M NaCl via a gradient elution. H_C_/A2 was further purified by gel filtration using a superdex 200 column into a final buffer of 50 mM Tris pH 7.4, and 150 mM NaCl. The purified protein was flash frozen in liquid nitrogen for storage at −20 °C until required for crystallisation.

### 4.2. X-ray Crystallography

Crystals of H_C_/A2 and H_C_/A2:GD1a were grown using the sitting drop vapour diffusion method at concentrations of 17 mg/mL and 8 mg/mL, respectively. For the latter, H_C_/A2 was incubated with 5 mM GD1a for at least 1 h prior to setting up crystallisation screens. H_C_/A2 crystals grew at 16 °C in 0.1 M Sodium acetate, pH 4.5, 22% *v/v* PEG smear broad (4.55% PEG 400, 4.55% PEG 500 MME, 4.55% PEG 600, 4.55% PEG 1000, 4.55% PEG 2000, 4.55% PEG 3350, 4.55% PEG 4000, 4.55% PEG 5000, 4.55% PEG 6000, 4.55% PEG 8000, and 4.55% PEG 10,000), whereas H_C_/A2:GD1a crystals grew at 16 °C in 0.2 M lithium citate tribasic tetrahydrate, 20% *w/v* PEG 3350. Crystals were mounted into a cryoloop and flash frozen in liquid nitrogen. Diffraction data were collected on I04 beamline at Diamond Light Source (Oxon, UK). A total of 7200 images were collected at 0.1° oscillation with exposure times of 0.01 s, for both H_C_/A2 and H_C_/A2:GD1a crystals. Data processing was carried out in DIALS [38] and both structures were determined by molecular replacement using PHASER [39] as part of the CCP4 package [40]. The H_C_/A2:SV2C (PDB: 5MOY) structure (excluding the coordinates for SV2C) was used as a search model for H_C_/A2 [23], and the refined H_C_/A2 structure was subsequently used as a search model for H_C_/A2:GD1a. Both structures were refined using REFMAC [41] and Phenix [42], with modelling performed in COOT [43]. The structures were validated using Molprobity [44] and PDB validation [45]. Figures were produced using CCP4mg [46].

## Figures and Tables

**Table 1:** X-ray crystallographic data collection and refinement statistics for the structures of H_C_/A2 alone and in complex with GD1a. Outer shell statistics are in parenthesis.

Beamline	I04	I04
Wavelength	0.9795 Å	0.9795 Å
**Protein**	**H_C_/A2**	**H_C_/A2:GD1a**
**Crystallographic statistics**		
Space group	P 2_1_ 2_1_ 2_1_	P 4_1_
Unit cell dimensions		
a, b, c (Å)	39.92, 100.79, 116.15	105.07, 105.07, 132.58
α, β, γ (°)	90, 90, 90	90, 90, 90
Resolution range (Å)	116.15–1.63 (1.66–1.63)	105.07–2.10 (2.14–2.10)
R_merge_	0.159 (5.530)	0.246 (3.916)
R_pim_	0.031 (1.087)	0.048 (0.847)
<I/σ(I)>	11.6 (0.7)	11.1 (0.9)
CC_1/2_	0.999 (0.299)	0.999 (0.400)
Completeness (%)	100.00 (99.90)	100.00 (100.00)
No. observed reflections	1,563,838 (77,009)	2,288,498 (4633)
No. unique reflections	59,567 (2930)	83,782 (4633)
Multiplicity	26.3 (26.3)	27.3 (21.9)
**Refinement Statistics**		
R_work_/R_free_	0.205/0.233	0.189/0.227
RMSD bond lengths (Å)	0.011	0.010
RMSD bond angles (°)	1.53	1.61
Ramachandran plot statistics (%)		
Favoured	96.00	95.00
Allowed	4.00	5.00
Outliers	0.00	0.00
Average B-Factors (Å2)	32.80	48.6
Protein atoms	32.52	44.98
Solvent atoms	36.94	46.24
GD1a ligand	N/A	84.2
No. Atoms	3606	7143
Protein	3372	6733
Solvent	234	333
GD1a	N/A	77
**PDB code**	**7Z5T**	**7Z5S**

## Data Availability

The atomic coordinates and structure factors of H_C_/A2 and H_C_/A2:GD1a were deposited in the protein data bank under accession codes 7Z5T and 7Z5S, respectively.
